# Thermal treatment alternatives for enzymes inactivation in fruit juices: Recent breakthroughs and advancements

**DOI:** 10.1016/j.ultsonch.2022.105999

**Published:** 2022-04-04

**Authors:** Muhammad Umair, Sidra Jabeen, Zekai Ke, Saqib Jabbar, Faiqa Javed, Muhammad Abid, Kashif-ur Rehman Khan, Yu Ji, Sameh A. Korma, Mohamed T. El-Saadony, Liqing Zhao, Ilaria Cacciotti, Clara Mariana Gonçalves Lima, Carlos Adam Conte-Junior

**Affiliations:** a Department of Food Science and Engineering, College of Chemistry and Engineering, Shenzhen University, 518060 Shenzhen, Guangdong, China; b Key Laboratory of Optoelectronic Devices and Systems, College of Physics and Optoelectronic Engineering, Ministry of Education and Guangdong Province, Shenzhen University, Shenzhen 518060, Guangdong, China; c National Institute of Food Science and Technology, University of Agriculture, Faisalabad 38000, Pakistan; d Department of Orthopaedics, Shenzhen University General Hospital, Shenzhen University Clinical Medical Academy, Shenzhen, Guangdong, China; e Food Science Research Institute (FSRI), National Agricultural Research Centre (NARC), Islamabad, Pakistan; f Institute of Food and Nutritional Sciences, Pir Mehr Ali Shah, Arid Agriculture University Rawalpindi, Pakistan; g Department of Pharmaceutical Chemistry, Faculty of Pharmacy, The Islamia University of Bahawalpur, 63100 Bahawalpur, Pakistan; h Lehrstuhl für Biotechnologie, RWTH Aachen University, Worringerweg 3, Aachen 52074, Germany; i Department of Food Science, Faculty of Agriculture, Zagazig University, Zagazig 44519, Egypt; j Department of Agricultural Microbiology, Faculty of Agriculture, Zagazig University, Zagazig 44519, Egypt; k Department of Engineering, INSTM RU, University of Rome “Niccolò Cusano”, Roma 00166, Italy; l Department of Food Science, Federal University of Lavras, Minas Gerais, Brazil; m Center for Food Analysis (NAL), Technological Development Support Laboratory (LADETEC), Federal University of Rio de Janeiro (UFRJ), Cidade Universitária, Rio de Janeiro 21941-598, Brazil

**Keywords:** Fruit juices, Enzymes, Polyphenol oxidase, Peroxidase, Non-thermal technologies, Quality

## Abstract

Fruit juices (FJs) are frequently taken owing to their nutritious benefits, appealing flavour, and vibrant colour. The colours of the FJs are critical indicators of the qualitative features that influence the consumer's attention. Although FJs' intrinsic acidity serves as a barrier to bacterial growth, their enzymatic stability remains an issue for their shelf life. Inactivation of enzymes is critical during FJ processing, and selective inactivation is the primary focus of enzyme inactivation. The merchants, on the other hand, want the FJs to stay stable. The most prevalent technique of processing FJ is by conventional heat treatment, which degrades its nutritive value and appearance. The FJ processing industry has undergone a dramatic transformation from thermal treatments to nonthermal treatments (NTTs) during the past two decades to meet the requirements for microbiological and enzymatic stability. The manufacturers want safe and stable FJs, while buyers want high-quality FJs. According to the past investigation, NTTs have the potential to manufacture microbiologically safe and enzymatically stable FJs with low loss of bioactive components. Furthermore, it has been demonstrated that different NTTs combined with or without other NTTs or mild heating as a hurdle technology increase the synergistic effect for microbiological safety and stability of FJs. Concise information about the variables that affect NTTs' action mode has also been addressed. Primary inactivates enzymes by modifying the protein structure and active site conformation. NTTs may increase enzyme activity depending on the nature of the enzyme contained in FJs, the applied pressure, pH, temperature, and treatment period. This is due to the release of membrane-bound enzymes as well as changes in protein structure and active sites that allow substrate interaction. Additionally, the combination of several NTTs as a hurdle technology, as well as temperature and treatment periods, resulted in increased enzyme inactivation in FJs. Therefore, a combination of thermal and non-thermal technologies is suggested to increase the effectiveness of the process as well as preserve the juice quality.

## Highlights

• Fruit juices (FTs) are important nutritional part of daily life.
• Demand for FJs processing with a certain safety standards urges nonthermal technologies (NNTs).
• NTTs can causes inactivate enzyme by conformational alteration.
• Food matrix, processing intensity, properties of enzymes and proteins present in FJs may affect NNTs efficiency.
• Combination of NNTs may cause synergistic enzyme inactivation effect.
• NNTs employment potentially ensures to maintain the FJs quality.

## 1 Introduction

Regulatory authorities demand FJs with a certain minimum level of quality and safety standards that can be considered as one of the most important concerns for food scientists and consumers, and strict requirements from the manufacturer’s perspective. In addition to adequate safety and quality, the retailer wants the processed FJs to be stable during storage [1]. The quality standards defines the quality of water for soft drinks should be free from any component that adversely effects the sensory (taste, odour and colour) and physicochemical properties of FJs. The organic matter should be < 1 mg/L, alkalinity and dissolved solids should be 50 mg/L and < 500 mg/L respectively. The sugar or sweetening agents such as glucose and fructose corn syrup (72°Brix), artificial sweeteners i.e., saccharin, and granulated sugar also affects the quality of FJs. Therefore, specific quality parameters (<1% arsenic content, < 0.02% ash contents) have been established for FJs [2], [3], [4]. To ensure the safety concerns of FJs, the microbial inactivation are the most challenging tasks of modern food processing. In NTTs, PEF, UV irradiation, HPP and ultrasonication have been extensively studied for deactivation of *L*. *innocua*, *S*. *aureus*, *E*. *coli*, and spoilage yeast while, the synergistic effects of NTTs are very promising for tackling the more resistant spores and species [5], [6], [7].

Thermal (conventional) methods are used to preserve the food [3], [8] and causes undesirable changes in food products due to the long term heat exposure results in the degradation of food quality. These methods can effectively decontaminate the food and inactivate the enzymes However; resulting compounds are chemical toxicants that are carcinogenic and harmful for human body. The amount and nature of toxicants depend on the type of thermal treatment used for processing and cooking food [9]. It can also cause the loss of water from food, oxidation of lipids that leads to the changes in the composition of fatty acids. The consumers' awareness regarding food quality and safety has been increased and they demand food that is free from microorganism, high nutritional and organoleptic properties with excellent mouth feel [10], [11].

In non-thermal technologies (NTTs), the food is treated at room temperature for a very short period (∼l min or less), maintaining its nutritional composition, and ensuring unchanged mouth feels, without damaging the product texture. There is no damage to food because heat-sensitive nutritious materials are intact in food without increasing the food temperature [3], [12], [6]. The NTTs that have been emerged over the past few decades are: irradiation (IR), ohmic heating (OH), pulsed electric field (PEF), high voltage electric field cold plasma (HVCP), ultrasonication (US), high hydrostatic pressure (HHP) processing etc., The significance of these technologies has been greatly increased due to their applications in enzyme deactivation that are responsible for the quality degradation and spoilage in various FJs [12], [13]. In the application of NTTs, the ohmic heating is used for the extraction of bioactivecomponents, enzyme inactivation with great retention of desired ingredients in FJs. It provides a rapid and uniform heat transformation to the FJ viscous and heat-sensitive products with less colour degradation [14]. PEF processing is applied directly to FJs in order to destroy the pathogenic bacteria, inactivation of enzymes, and recovery of bioactive components, freezing and structure modification. For enzyme deactivation the specific electric field intensity is used 15–60 kV cm^− 1^ [15], [16], with specific energy 110–240 kJ kg^− 1^ for various FJs. However the inactivation of most undesirable enzymes remains ineffective due to their requirement for high intensity electric field [17], [18]. Ultrasonication is used for the extraction, synthesis, and preservation of FJs. The combined effect of PEF and US is a novel non-thermal method, which has shown its capability to enhance the quality and safety of FJs with small nutrient losses. These methods reduced the processing time, high through-put, less energy inputs, and are eco-friendly [5], [19], [20]. Cold plasma is a promising, novel non-thermal food processing treatment in which reactive nitrogen species (RNS) (N_2_^+^, N•, NO•, NO etc.) and reactive oxygen species (ROS) (OH^–^, •OH, H_2_O_2_, O_2_^–-^) are generated due to the feed gas ionization in a high electric field [21]. It induced the microbial and enzyme inactivation and reduced the anti-nutritional components and contaminants in foodstuffs. Therefore, it enhances the quality and stability by keeping heat-sensitive nutrients [4], [22]. The HHP processing is non-thermal energy-efficient technology used forthe FJs treatment, killing the microorganisms and inhibiting the oxidative enzymes, while masking the other chemicals and the quality degrading mechanisms in FJs [23], [24]. In this process, 100–1000 MP pressure is applied to the FJs with or without heat. HPP works on force compression principle which applied to surrounding fluid of the product. Pressure has a partial effect on covalent-bonds of low-molecular-mass compounds such as pigments, vitamins, and volatile substances as compared to high-molecular-mass molecules including enzymes or proteins. The three dimensional structure of enzymes is stabilised due to covalent/non-covalent interactions therefore, series of reactions involve in the destruction and formation of new linkages, folding and unfolding thus causes to bring changes in natural structure of enzymes [3], [9], [23], [25].

Enzymes naturally present in FJs, including peroxidase (POD), polyphenol oxidase (PPO), pectin methyl esterase (PME), and lipoxygenase (LOX), causesquality degradation FJs. These enzymes catalyze the phenolic components to o-quinones and melamines after polymerization, resulting in the development of off-flavours. For instance, POD and PPO are responsible for the oxidation of a large number of compounds in the presence of hydrogen peroxide (H_2_O_2_), such as the phenolics, contributing to the enzymatic-browning [14], [26], [27], [28]. On the other hand, PME is a hydrolytic enzyme of the cell wall and is also used for the FJs clarification process to reduce their viscosity. However, in the processing of de-pectinate and clarified FJs, it is important to leave these enzymes to eliminate the starch and pectin [29]. However, in some purees and juices that contain a suspended pulp, like citrus juices, it is important to inhibit the activities of endogenous enzymes to avoid the cloud loss in the final product. The PME can also lower the FJs viscosity, contributing to reduce the consumer acceptance and the FJs overall quality. PME hydrolyses the pectin in FJs, causes the instability of cloud and decreases the viscosity of FJs by the degradation of pectin chain. PME is present in all citrus fruits as a bounded cell wall enzyme that forms a complex with pectin and induces the electrostatic linkages. During FJs extraction, PME is released, hydrolysing the pectin and changing it gradually into the pectic-acids and tolowmethoxy pectin. These insoluble compounds interact with calcium ions [30], [31], induces the precipitation of the pectin and loss of cloud in FJs [9], [32], [33]. Another FJs natural enzyme, namely LOX, is responsible for the production of volatile flavours and free radicals in many FJs, causing colour and nutritional losses through the oxidation of polyunsaturated fatty acids (PUFA) into hydroperoxides. Therefore, in FJs, the inactivation of endogenous enzymes is very important for the production of high-quality products [22], [24], [34], [35].

It has been reported that the degree of enzyme inactivation varies depending on the food type and processing conditions [36], [37], [38], [39], [40], [41], [42]. For same fruit or cultivar, the processing time and temperature can be different depending on the type of technology used for enzyme inactivation. For example, in apple puree, 80% inhibition of PPO occurred after 3 min, in thermosonication treatment at 72 ℃ and 460 W/cm^2^ and for HPP-treatment 71℃, 50 min were selected at 600 MPa. The combination of ultrasound and HPP with thermal treatment offers an advantage in terms of PPO inactivation. Even though, the different forms of same fruit like puree, juice, and concentrate can also affect the inactivation of PPO for similar treatments [6], [43], [44].

In addition to microbial safety and enzyme stability, the consumers also demand minimally processed products with quality equivalent to fresh produces. In this context, the application of NTTs attains a key role. A comprehensive review about the effect of all NTTs on FJs enzymes inactivation has not been reported yet. The present review is to focus on the influence of all non-thermal processing techniques on FJs and provide discussions about the general FJs spoilage, an overview on NTTs, fundamental procedures, factors affecting NTTs, enzymes inactivation mechanisms, and NTTs effect on various FJs quality attributes.

## 2 Irradiation (IR)

In FJs, the IR processing is an established method to inactivate their enzymes. It involves the use of ionizing radiations with sufficient energy to emit the electrons from the product molecules. The general standards of Codex Alimentarius allow the use of food irradiations including the radioactive isotopes of cesium-137 (Cs ^137^) and, cobalt-60 (Co ^60^), as well as high energy electrons beam [45]. These electrons are produced by a machine, applying maximum energy to generate β-rays of 10 million electron-volts (MeV, while X-rays are produced by applying the energy of 5 MeV (Fig. 1a) [45], [46]. These rays have sufficient energy to convert the electrons into ions of food molecules which are electrically charged particles and reactive oxygen species (ROS). During IR processing, the rays break down the ionized molecules and chemical bonds present in the enzymes [47]. The dose of irradiation acquired by the food during IR processing is measured in kilo grays (kGy), and one Gy equals 1 J of energy absorbed by 1 kg of food. The penetration power of gamma and X-rays into the food matrix is different: the X-rays penetration through the water equivalent matter is 23.00 cm while for the gamma rays 3.90 cm [48].

**Fig. 1a:**
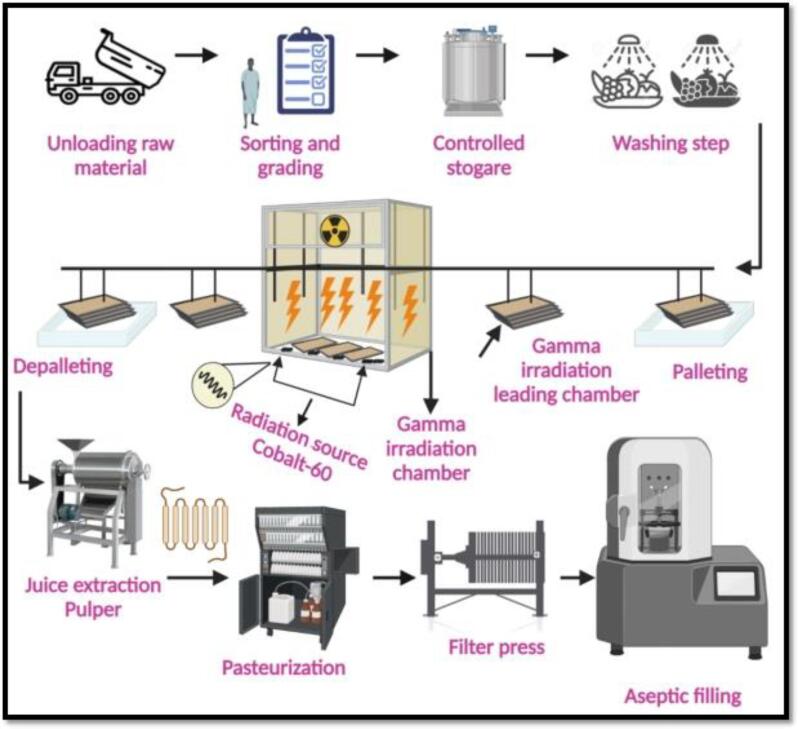
Flow diagram of the fruit juices processing steps using a gamma-radiation-assisted extraction system. Adapted from Kalaiselvan, Sugumar [46] with permission.

The ultraviolet light electromagnetic spectrum includes radio waves, microwaves, infrared radiation, X-rays visible light, and γ-radiations. In high-energy UV radiations, the electromagnetic spectrum in the 100–400 nm range, categorized as UV-A, UV-B, and UV-C from 320 to 400 nm, 280 to 320 nm, and 200 to 280 nm, respectively, are used for the treatment of different food products [49], [50]. In particular, the UV radiation of 254 nm is extensively used in FJs during IR processing [51]. Several studies have been carried out on the UV-radiation of FJs to study its effect on the microbial inactivation. However, few studies reported the UV radiations effect on the enzyme activities that are responsible for browning reactions in FJs containing polyphenolic compounds (Table 1). The PPO, POD, and PME activities in apple juice were monitored when exposed to a 400 W mercury lamp that emits radiations of 250 and 740 nm [51]. Results revealed that the PPO activity was inhibited after 100 min, that of POD completely inactivated in 15 min, and that of PME (in one variety) in 40 min after the exposure to UV radiation [51]. Moreover, authors observed no modifications in pH, total phenolic content, sugars, and soluble solids in the treated and controlled apple juice samples [51].

**Table 1:** IR processing effect on the enzyme activities in various fruit juices.

**Fruit juices**	**Dose/fluency (mJ/cm^2^)**	**PPO (residual activity)**	**POD (residual activity)**	**References**
Apple-juice	NC	99.51	97.00	[78]
Apple-juice (Sparkling)	NC	0.00	0.00	[126]
Grape-juice (Dauphine)	NC	20.00	0.00	[127]
Pear-juice	NC	0.00	0.00	[128]
Orange-juice	58.21	75.00	97.00	[129]
Apple-juice (Clear)	12,483	10.00	0.00	[130]
Nectarine-Juice	NC	60.00	40.00	[26]
Clear apple-juice	72.00	70.00	NA	[131]
Grape-juice	30.10 kJ/L	60.90	NA	[132]
Apple-juice	8.10 kJ/L	86.10	NA	[133]
Apple-juice	1.50 kJ/L	114.50	NA	[134]
Mango nectar-juice	108 kJ/L	19.00	NA	[125]
PPO: polyphenoloxidase; POD: peroxidase; IR: irradiation				

The action mode of ionizing radiations on enzymesis originated from the biochemical changes in the biological system (Fig. 1b) [46]. The exposure of irradiation, x-rays gamma rays, and high-speed electrons induces chemical changes in the enzyme molecule atomic groups, with consequent inhibition of enzyme activities in FJs [52]. It has been reported that the gamma rays and x-rays exposure up to 100 Gy could create a significant enzyme reduction activity in FJs [53]. Moreover, the x-rays exposure could inactivate enzymes that contain the active sulfhydryl group or thiol group, due to the oxidation of the sulfhydryl groups or thiol group, with disulfides inhibition [54].

**Fig. 1b:**
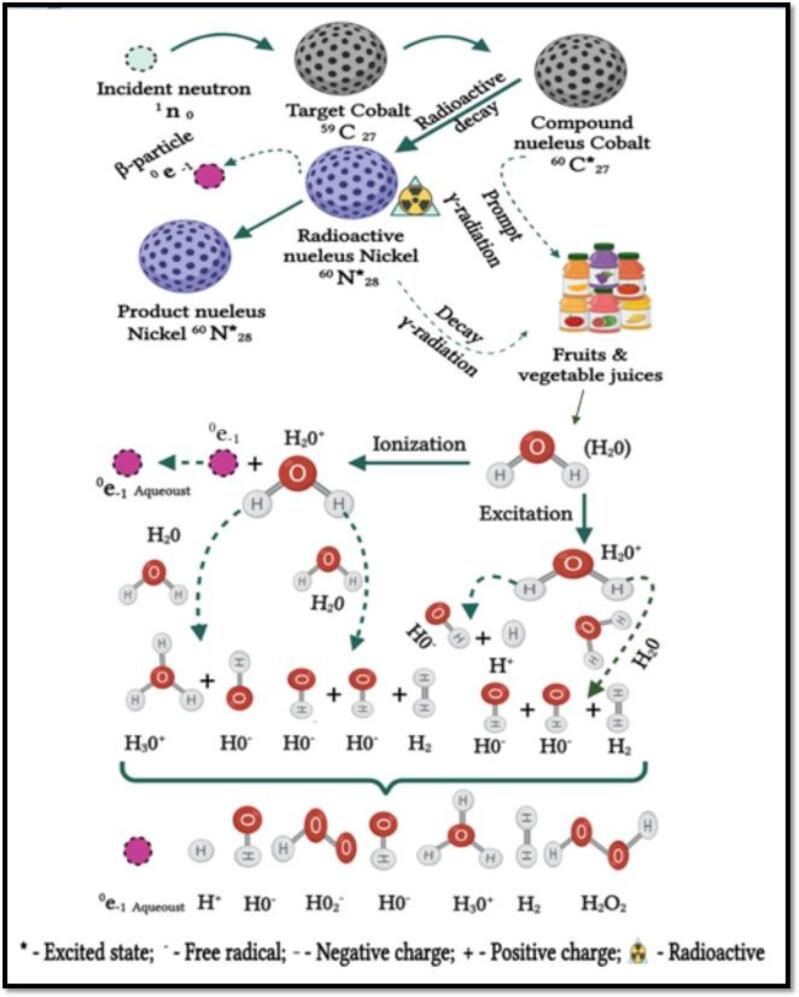
Gamma-irradiation theory of water molecules radiolysis in fruit juices. Adapted from Kalaiselvan, Sugumar [46] with permission.

## 3 Ohmic heating (OH)

The OH processing consists in the passing of an alternating current through liquids and food substances. It presents an advantage over the traditional thermal processes, being able to extend the shelf life of pomegranate, melon, apple, and orange juices [55]. This NTT works faster with 4.6 to 5.3 times lower energy consumption than that of the conventional heating [55]. Furthermore, OH processing has revolutionized the FJs industry for its effect on FJs bioactive components, sensory properties, enzyme and microbial deactivation, and loss of the physicochemical attributes [56]. Moreover, it plays a significant impact on dehydration or concertation, sterilization, blanching, pasteurization, and recovery of phenolic components and their antioxidant potential [57]. Several studies have been greatly focused on developing OH processing as an established and economical (being based only on electricity) alternative to the thermal technologies, including controlled agitation processing, reciprocation processing, radio-frequency, and microwave heating [58], [59].

**Fig. 2:**
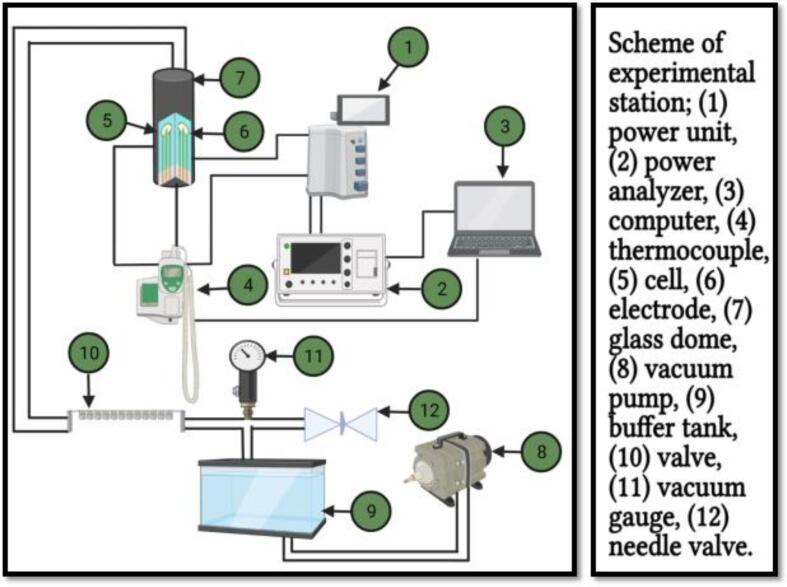
Schematic diagram of the ohmic heating (OH) processing system; (1) power unit, (2) power analyzer, (3) computer, (4) thermocouple, (5) cell, (6) electrode, (7) glass dome, (8) vacuum pump, (9) buffer tank, (10) valve, (11) vacuum gauge, (12) needle valve. Adapted from Fadavi and Salari [60] with permission.

Concerning the OH processing action mode, it is based on electromagnetic methods such as, i.e., radioactive dielectric, capacitive dielectric, radioactive magnetic, and inductive heating ones (Fig. 2) [60]. Moreover, OH processing is similar, to some extent, to the microwave heating systems at different frequencies [61]. In the direct resistance OH processing, liquid food and solid substances are simultaneously heated by means of passing an electric current through the samples, whereas in continuous OH processing, a generator and a power supply are used to generate the electric current [14], [62]. The electrodes are in direct contact with the food material and pass the current through the food particles [61]. The distances among the electrodes are so adjusted, to generate the optimum electric field [61]. The heat generation and the overall enzymes inactivation mechanism are strongly affected by the electrical field strength and the residence time [55]. A greater production of grape juice up to 50–70% was noted with ohmically heated grape mash than conventionally heated one. This increase in the yield has been recorded: 10.14% for OH and 5.23% for conventionally heated grape mash compared with the untreated group (control) [6], [63], [64]. The generator produces the electrical current that passes from the first electrode and flows through the food matrix that lies between the electrode gaps < a food particle resists the electric-current flow and induces the instant volumetric heat generation by following the Joule first-law of heating. The current continuously passes to the second electrode, then back to the power supply to close the circuit. The insulator caps near the electrodes control the entire system [65], [66].

The OH processing is efficient to inhibit the PPO and POD activities at a lower temperature with a short time without damaging the FJs quality [67]. Some studies reported that the PPO activity in sugarcane juice was decreased up to 97.80% at 90 °C for 5 min when OH processing of 32 V/cm was carried out [68]. Another research group of Bhat, Saini [70] investigated the OH processing and thermal treatment effect on various quality features such as colour and total phenolic content (TPC) of bottle guard juices at 60 to 90 °C for 1 to 5 min. The authors evidenced better colour retention and higher TPC valuesas primary OH processing outcomes, due to the higher enzymes deactivation than in thermally treated (90 °C for 5 min) juice samples [69].

A study reported that the grape juice preservation was increased by increasing the voltage gradient duringthe OH processing, demonstrating a higher PPO inhibition at 40 V/cm than at 20 and 30 V/cm. It could be possible due to the rapid electric conductivity increment that induces greater PPO inhibition at a higher voltage gradient. Furthermore, at 60 °C with a constant voltage gradient during OH processing, a non-significant increase in PPO activity was observed [70]. Baysal, Demirdöven [64], studied the OH processing application in the grape juice yield by grape pulp hot pressing.

## 4 Pulsed electric field (PEF)

The PEF processing has been used as an emerging technology in the food industry over the last few decades for the processing of various FJs such as orange, apple, carrot, and tomato juices. It is used for the high-quality food products as it is efficiently inhibits the activities of different enzymes, including POD, PPO, PME, and LOX, in FJs and several other beverage products (Table 2). In comparison to the microbial inactivation, high-energy inputs are required for enzyme inhibition. At low intensity, PEF processing can be also used for controlling the enzymes activities [71], [72].

**Table 2:** PEF processing effect on the enzyme inactivation in fruit juices.

**Fruit juices**	**Enzyme**	**PEF system**	**PEF parameters**	**Reduction rate**	**References**
Apple-juice	PPO	Bench-scale and rectangular shape, bipolar-pulse, 0.64 cm gap	38.50 kV/cm, 50 °C combined with 300 pps	70%	[135]
Apple-juice	PPO, POD	P1: Pilot plant scale P2: Bench scale	P2:40 °C PEF P1: 60 °C PEF (100 kJ/kg, 30 kV/cm)	P1: 48% for PPO P2: 100% for PPO and POD	[79]
Orange-juice	PME	Pilot plant scale, co field flow-tubular and PEF treatment chamber, the electrode gap is 1.0 cm with stainless-steel tubular-electrode	35 kV/cm, 59 μs treatment time, 1.4 pulse width, 600 pps, 98 mL/s	88%	[136]
Orange-juice	PME	Co-field flow-tubular PEF treatment chamber system, electrode gap: 0.2 cm Stainless-steel electrode	20–35 kV/cm, 2.2 or 2.0 pulse width, 700 pps, 0.31, 0.42, mL/s	90.2%	[136]
Tomato-juice	PME	Gene-electroporator (Bio Rad Laboratories)	24 kV/cm, 800ls of treatment time with exponential decay	93.5%	[137]
Carrot-juice	POD	Continuous flow bench-scale PEF, co-field Flow-treatment chamber, 0.29 cm gap, square-wave bi-polar pulse	35 kV/cm for 1.000ls, 6ls pulse width at 200 Hz.	73.2%	[138]
White Grape-juice	POD, PPO	Bench scale, co-field flow-treatment square-wave bi-polar pulse	25–35 kV/cm at 200–1,000 Hz, 1–5 ms treatment	50% POD, 100% for PPO	[139]
PPO: polyphenoloxidase; POD: peroxidase; PME: pectinmethylesterase; PEF: pulse electric field					

In PEF processing, an electric field is applied instead of heat, high and short voltage pulses (10–80 kV/cm, lasting within seconds from micro to milliseconds) are connected among two electrodes directed towards the projected foodstuff [73]. A PEF processing consists of a monitoring and control system, high electrical power source, pulse initiator, an assembly room (treatment chamber), a cooling scheme to check and control the heat escalation, compartments of raw and treated foodstuff [74]. An energy input of 10–20 kJ/kg, for pre-treating to raise the temperature 40 ℃, is usually required in a typical PEF processing [75], [76]. And, therefore, the resulted product is a minimally processed juice. This type of product was characterized as pasteurized and fresh juice to meet the consumers’ expectations of freshness and shelf life.

A study reported the influence of PEF processing on freshly prepared apple juice in the PPO and POD deactivation. A study revealed that the reduction in the POD and PPO activity up to 68% and 71%, respectively, was achieved when a preheating (50 ℃), and a PEF processing (at 40 kV/cm for 100 μs) were applied together [77], [78]. This level of POD and PPO inhibition was significantly greater (P < 0.05) when compared with conventionally pasteurized juices, characterised by a POD and PPO inhibition of only 48% and 46%, respectively. Schilling, Schmid [80], also investigated the PEF processing influence on the apple juice quality, reporting that the PPO and POD complete inhibition was obtained when the PEF processing was combined with preheating process of juices to 60 °C. They observed the PPO deactivation up to 48% at 40 °C applying 30 kV/cm, 100 kJ/kg as optimal PEF process parameters [79].

As enzymes are proteins in nature, the PEF processing action mode to inhibit the enzymes activities involves the alterations of the secondary structure (α-helix and β-sheets), spatial conformation changes in the tertiary structure, and modifications in the arrangements and number of protein subunits in the quaternary structure, therefore, losing the enzymes functionality (Fig. 3a) [80].

**Fig. 3a:**
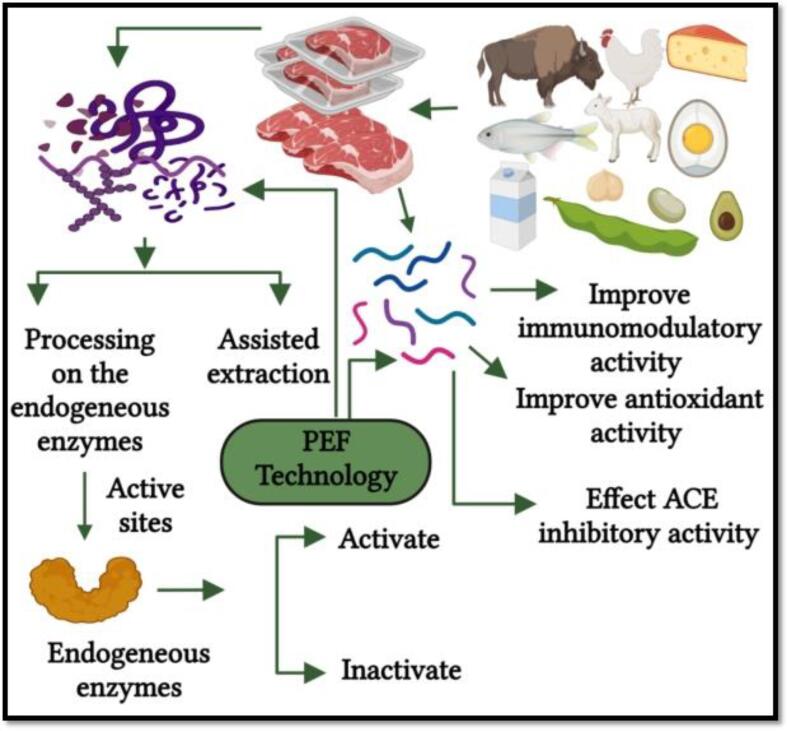
Pulsed electric field effect on the enzyme deactivation. Adapted from Zhang, Sun [80] with permission.

The PEF processing principle is based on two fundamental procedures, i.e. an electric-breakdown and electroporation (electric pulse used to form the temporary pores in the cell membrane), both coordinated to destroy the microorganisms and inactivate the enzymes [81]. The electric energy is uniformly transferred to whole food products due to the presence of charged particles [36], [82]. The PEF processing efficiency depends on several components, including intensity, field strength, the food particles conductivity, pH and temperature, the involved enzymes and pathogen nature, the pulse rate and amount of energy, the time interval, and the polarization (Fig. 3b) [74], [83], [84]. No studies have been reported the chemical changes induced in the enzymes primary structure by the PEF process [85]. However, the PEF processing parameters and conditions must be optimized to obtain desired enzyme inactivation [86].

**Fig. 3b:**
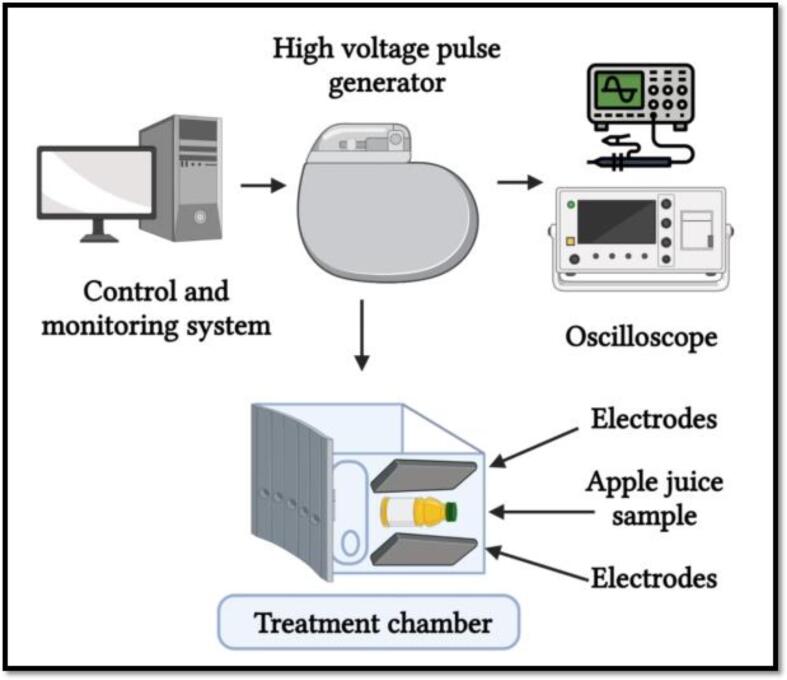
Pulsed electric field schematization. Adapted from Dziadek, Kopeć [83] with permission.

## 5 Ultrasonication (US)

Among NTTs, US is widely employed in the FJs industry [87]. Low-energy US processes are used for non-invasive purposes with sound intensities < 1 W/cm^2^ and frequencies > 100 kHz [88]. These ultrasound waves are applied during the fermentation by the imitation of living cell activity through degassing, for the monitoring of physicochemical properties such as composition, particle size, and flow rate (Fig. 4a) [27]. High-energy US, characterised by sound intensities < 1 W/cm^2^ and frequencies from 18 to100 kHz, are particularly employed for enzymes inactivation [89]. Furthermore, when US processing is combined with heat or pressure, it has been found to be greatly effective, particularly for FJs and beverages [44], [90].

**Fig. 4a:**
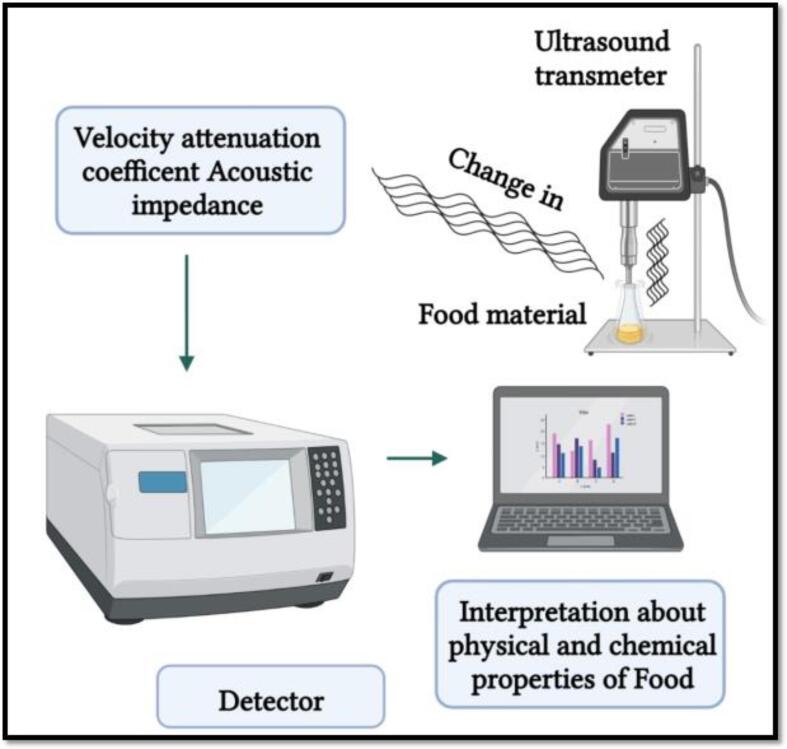
Represents low power ultrasound principles. Adapted from Dolas, Saravanan [27] with permission.

Some studies have reported the efficacy of US processing (Table 3), particularly in combination with non-thermal and thermal processing methods such as the high pressure and pH differences application [89]. On the basis of the frequency ranges, it is possible to identify two main US categories: high-intensity US (100–200 kHz, >1 W/cm^2^) and low-intensity US (>100 kHz and 0–1 W/cm^2^) [91], [92]. Low-intensity US waves are commonly used for the non-destructive investigation of the physicochemical changes in compounds during the food processing [93].

**Table 3:** US processingeffect on the enzyme inactivation in various fruit juices.

**Fruit juices**	**Ultrasonic System**	**Effect on enzyme deactivation with optimal conditions**	**References**
Orange-juice	System-A, D-19 mm and UP- 1500 Watt	• 62% PME inactivation at optimal conditions F-20 kHz, TI-20 min, AD-1.0 W/cm ^2^	[140]
Orange-juice	System-Aa, D-3 mm and UP-200 Watt	• 91% PME inactivation at optimal conditions F-24 kHz, TI-9.8 min, AD-80 W/cm^2^, T-63 °C	[141]
Mosambi juice	System-B, UP-400 Watt	• 96.8% PME inactivation at optimal conditions F-50 kHz, TI-20 min, AD-400 W/cm^2^, T-80 °C	[142]
Pineapple-Juice	System-A and D-13 mm and UP-500 Watt	• 20% PPO inactivation at optimal conditions F-19 kHz, TI-10 min, AD-376 W/cm ^2^	[143]
Apple-juice	System-Aa, D-13 mm and UP- 750 Watt	• 93.83%, 91% and 92.91% PPO, POD and PME inactivation at optimal conditions F-20 kHz, TI-10 min, AD-0.3WmL^−1^, T-60 °C	[144]
Pear-juice	System-A, D-12.7 mm and UP–750 Watt	• 95.6%, 96.73%, and 98.08% POD, PME, and PPO inactivation of at optimal conditions F-20 kHz, AL-70%, T-10 min, T-65 °C	[145]
Grape-juice	System-B, UP-420 Watt	• 91%, 90%, and 89% PME, PPO, and POD inactivation at optimal condition F-28 kHz, TI-60 min, AD-294 W/Cm ^2^, T-60 °C	[44]
Purple cactus pear- juice	System-Aa, D-13 mm, UP-1500 W	• PME activity decreased at F-20 kHz, AL-80%, TI-25 min, T-50 °C	[146]
Soursop-juice	System-A, D-1.3 cm, UP-500 Watt	• 14.73% PPO inactivation of PPO at optimal condition F-19 kHz, TI-9 min, AD-330 W/cm ^2^	[147]
Bayberry-juice	System-A, D-13 mm, UP-600 Watt	• 90% POD and PPO inactivation at optimal condition F-20 kHz, TI-6.7 min and 2.5 min AD-452 W/cm^2^, AL 100%	[148]
PPO: polyphenoloxidase; POD: peroxidase; PME: pectinmethylesterase; US: ultrasonication			

The US processing action mode related to the enzyme inactivation is based on the cavitation effects that originate during the food processing, including evolution, inception, and implosion or disintegration of small gas bubbles in a target food product. These cavitations consist in a mechanism of creation of high-power sound waves due to the formation of vapour or gas bubbles that are continuous and violently and rapidly implode in the sample solution. This implosion occurs at extremely high temperatures up to 1000 ℃ and high pressures from 50 to 500 MPa [88], [94]. This cavitation process originates due to the external stress which has been found to be very effective to bring the conformational secondary and tertiary structures changes through hydrogen bonds cleavage (Fig. 4b) [27], [95]. Moreover, the cavitation also produces free radicals due to the water hemolytic breakdown [96]. This results in free radicals of hydroxyl (OH^–^) and hydrogen (H^+^) species, able to react with free amino acids, responsible for the proteins and enzymes destabilization, leading to alterations in the biological compounds activity [88]. High-intensity US waves can be used to destroy the microorganisms and inhibit the enzyme activity by altering the proteins secondary structure (Fig. 4c) [27], thus leading to changes in their nutritional and functional properties, as highlighted earlier [97].

**Fig. 4b:**
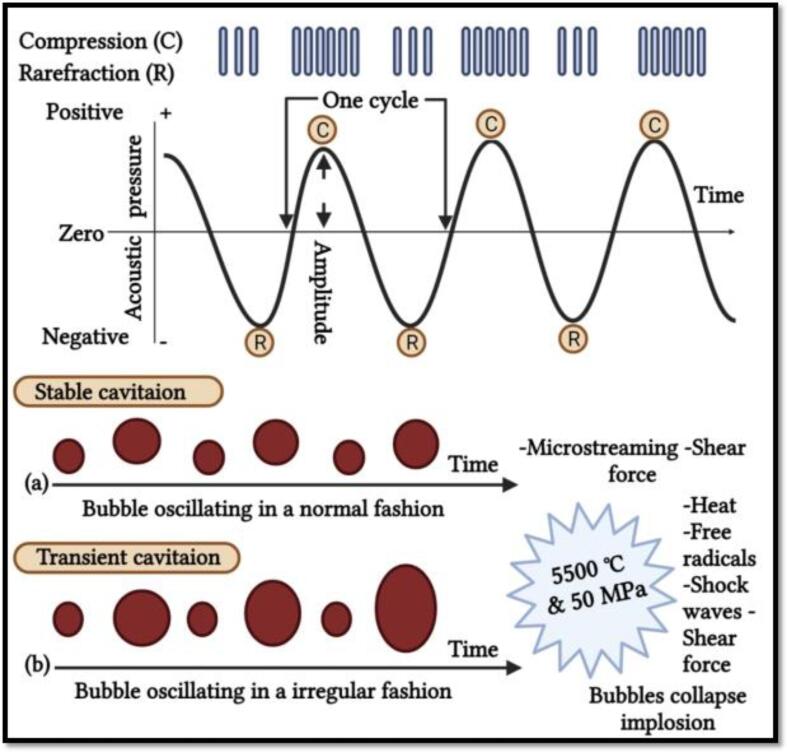
Cavitation mechanisms for: (a) stable cavitation, (b) transient cavitation. Adapted from Dolas, Saravanan [27] with permission.

**Fig. 4c:**
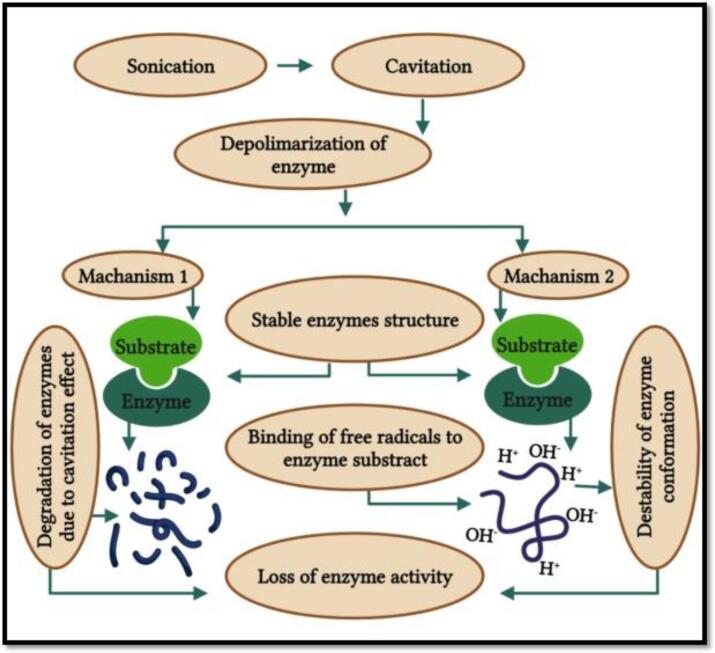
Represent the enzyme inactivation mechanism of FJs by ultrasound. Adapted from Dolas, Saravanan [27] with permission.

## 6 High voltage electric field cold plasma (HVCP)

In recent years, HVCP processing has been extensively used as a NTT for the enzymes inactivation in FJs [7], [87], [98], [99], [100], [101], [102]. It is based on protons, electrons, atoms, and ions in their excited and ground states. In function of the electron’s temperature, plasma is divided into high-temperature, i.e., Te (electron temperature) ¼ 10^6^ to 10^8^ K and low-temperature Te ¼ 10^4^ to 10^5^ K plasma [103], which is further classified into non-thermal and thermal plasma, on the basis of the thermodynamic equilibrium [104]. Non-thermal plasma produces the non-equilibrium thermodynamic effect in which Te can reach about 10^4^ K, which is far greater than the whole gas temperature. Therefore, the HVCP system can be retained at lower temperatures and is well known as cold plasma [105], [106].

The HVCP is produced by supplying an adequate energy amount to a neutral gas, causing its ionization, and, thus producing the chemically active components, such as charged particles, non-excited or excited molecules, UV radiations, and free radicals (e.g., reactive oxygen species (ROS), and reactive nitrogen species (RNS)) [87], [101], [102], [107]. The energy used to produce the plasmas is classified as alternating current (AC) discharges and direct current (DC) discharges, which are operated in pulsed mode or continuously [98], [108].

The HVCP plays a significant role in lowering the nutritional losses that can alter the macro-molecules, thus expanding its applications in the FJs industry [109], [110]. In particular, extensive HVCP applications have been reported for the pathogenic microorganisms and enzymes deactivation [98], [111], [112], being the HVCP major advantage the FJs processing at ambient temperature (Fig. 5a, Fig. 5b) [7], [99].

**Fig. 5a:**
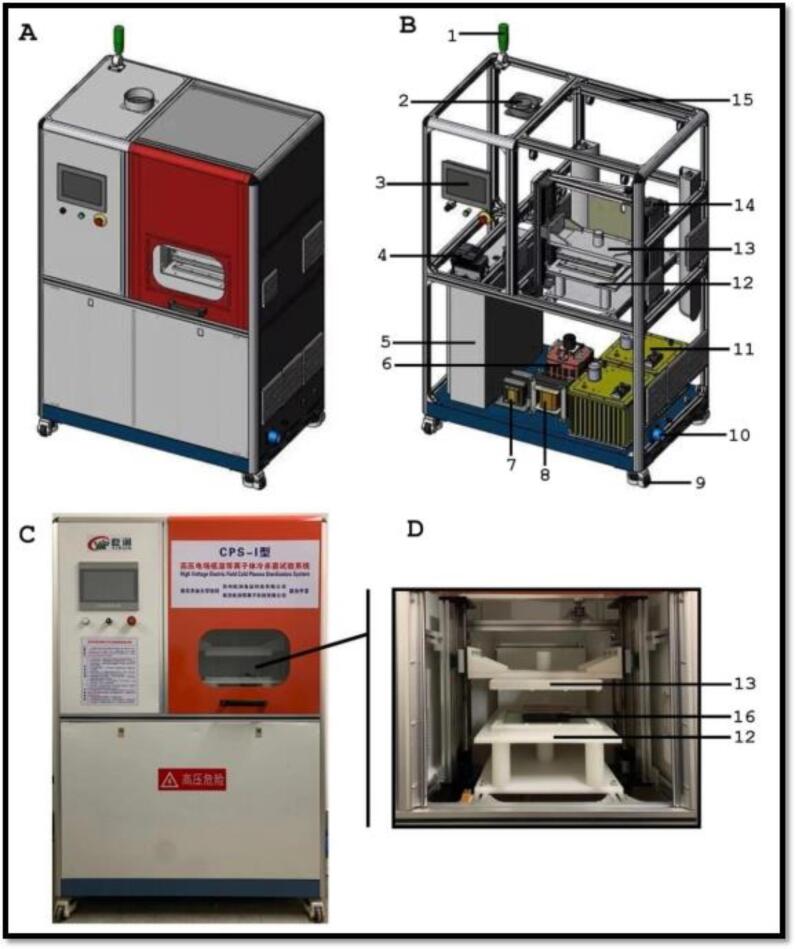
(**A**) Schematization of DBD-cold atmospheric plasma (CAP) machine; (**B**) Schematization of a DBD-CAP machine with the indication of its components; (**C**) Picture of a DBD-CAP machine; (**D**) Treatment chamber of a DBD-CAP machine. Legend; 1, Indicator; 2, Exhaust fan; 3, Touch screen control panel; 4, Frequency converter; 5, Power distribution box; 6, Electric voltage regulator; 7, Inductance 1; 8, Inductance 2; 9, Wheels; 10, Power cable inlet; 11, High voltage transformer; 12, lower electrode; 13, upper electrode; 14, lifting mechanism; 15, Frame; 16, Sample position. Adapted from Nasiru, Frimpong [99] with permission.

**Fig. 5b:**
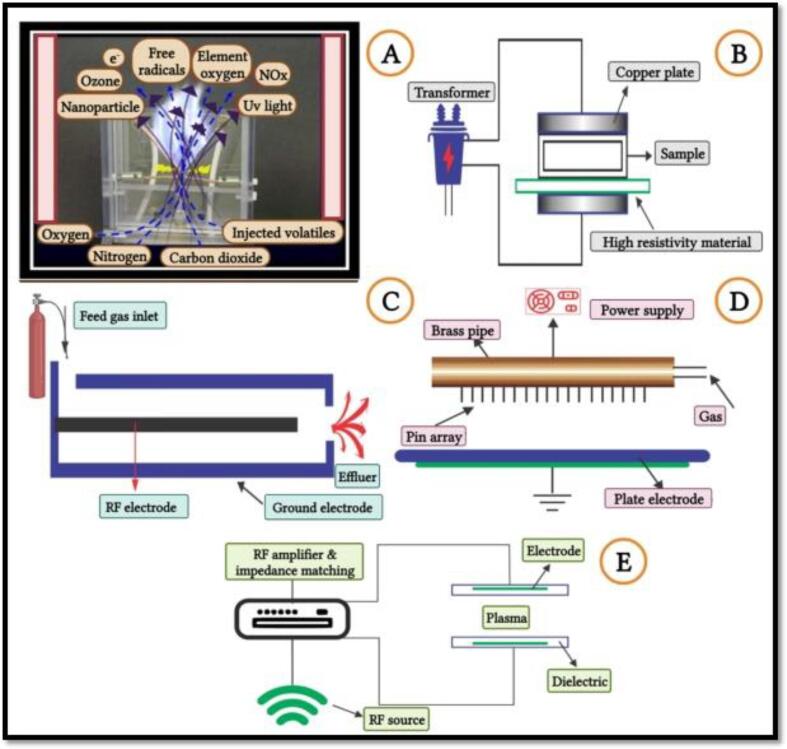
Description of different sources used for the plasma generation. (A) plasma generation-ionization processing, (B) resistive barrier discharge (RBD), (C) atmospheric pressure plasma jet (APPJ), (D) corona discharge system, (E) configuration of the DBD-BASED diffuse glow discharge atmospheric pressure. Adopted from Umair, Jabbar [7] with permission.

The HVCP action mode in the enzyme deactivation is based on its effect on the enzymes secondary structure (Fig. 5c) [113]. The ROS and RNS species can induce the enzyme inhibition by preventing the binding of coenzyme/substrate to their subsequent catalysis that occurs due to the conformational changes in the enzymes active site [53]. During HVCP processing, under the electric field effect, the primary target of HVCP is α-helix and β pleated sheets of enzyme’s protein [114]. Radical exposure of these proteins causes chemical and physical changes such as the cleavage of backbone or fragmentation, side-chain oxidation, unfolding, cross-linking, changes in conformation, and hydrophobicity, thus altering the susceptibility to proteolytic enzymes [115]. Some authors reported that the α-helix structure of POD and PPO disrupted from 34.90% to 5% and 36.90% to 17.80% while β-sheets improved from 15.60% to 39.90% and 15.20% to 29.40%, respectively during HVCP processing when treatment time was increased up to 360 s. This change in the enzymes secondary structure occurs due to the protein polymers/plasma-induce reactive species interaction [7], [98].

**Fig. 5c:**
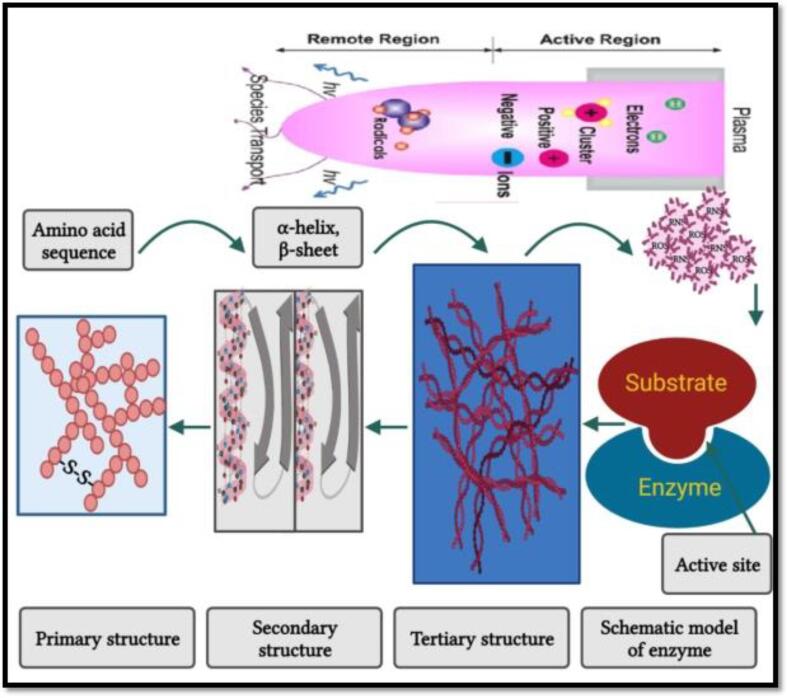
Schematization related to cold plasma treatment effects on the enzyme conformation, reactive oxygen species (ROS); reactive nitrogen specie (RNS). Adapted from Han, Cheng [113] with permission.

A researcher [116], has reported the effect of HVCP on the cloudy apple juice quality parameters, maintaining optimal conditions with a power input of 10.5 kV for 5 min. The physicochemical properties and enzyme inhibition were dependent on the treatment duration: a significant PPO activity reduction was achieved after 5 min and a complete PPO inactivation after 24 h. A remarkable pH decrement was noticed due to the reactive species development after HVCP processing [116], whereas the juice colour was also improved after long treatment. However, the treated FJs were lighter than untreated juice samples that were maintained during the storage period (Table 4).

**Table 4:** HVCP effects on the enzymes inactivation in different fruit juices.

**Fruit Juices**	**Enzyme**	**Plasma**	**Results**	**References**
Apples	PPO	DBD, 15 kV, 12.7 kHz, 10–30 min, Air, 1.5 m/s	• Linear inactivityreductiondue to the treatment time • Residual activity of 42%, 68%, and 88% after 30, 20, and 10 min of treatment	[107]
Apples	PPO	DBD, 150 W, 15 + 15, 30 + 30 min, Air, 1.5 m/s	• A significant decrease in superficial browning not proportional to the treatment time • Varying impact on the PPO activity • Cultivar dependent strict effects	[149]
Melon	POD	DBD, 15 kV, 12.5 kHz, 15 + 15, 30 + 30 min	• Air residue activity of 91% and 82% after treatment for15 + 5 and 30 + 30 min, respectively	[150]
Melon	PME	DBD, 15 kV, 12.5 kHz, 15 + 15, 30 + 30 min	• not effective 15 + 15 min air treatment • 94% residual activity after 30 + 30 min treatment	[150]
Carrot	PPO	DBD, 70 kV, 3 min, air	• 33–43 % residual activity during 28 days storage at 4 ℃	[121]
PPO: polyphenoloxidase; POD: peroxidase; PME: pectinmethylesterase; HVCP: high voltage electric field cold plasma				

## 7 High hydrostatic pressure (HHP)

The HHP processing is a another NTT that extensively used to preserve the quality of different food products including liquid food (bottled), high-moisture solid food (vacuum packed), and medium or low moisture solids and semisolid foods (vacuum packed) [117]. To lower the post-contamination risks, these products are packed before the processing, placed in baskets, then lowered into the vessel [9], [23], which is filled with water. The HHP processing (800 MPa) is uniformly spread in the vessel in order to assure the thorough processing of the product [24], [35], [118], [119], [120].

**Fig. 6:**
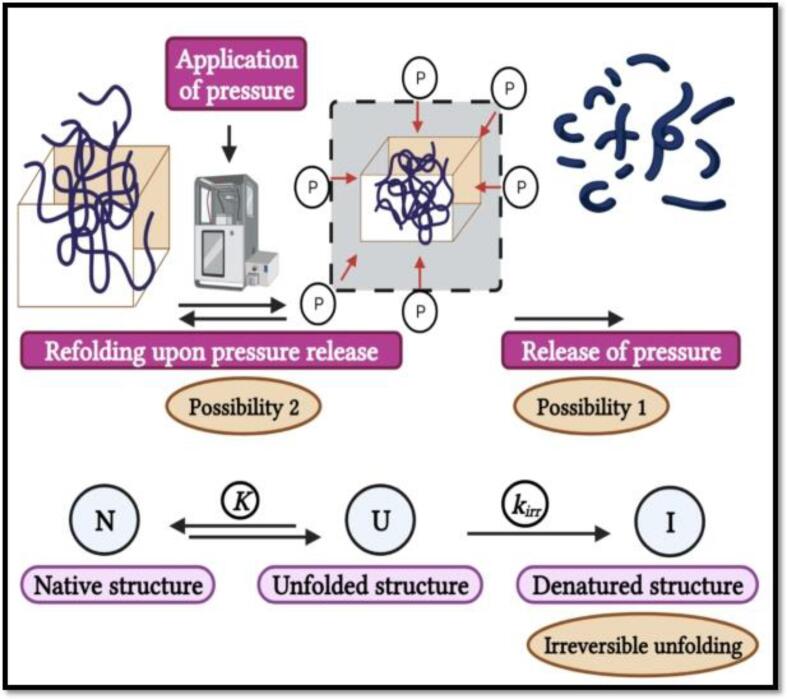
Hypothesised mechanism of high-pressure effect on the enzyme conformational structure. Adapted from Chakraborty, Kaushik [24] with permission.

The effect of HHP processing on the enzyme inactivation involves the unfolding of the endogenous enzymes promote in structure, changes in the cell membrane fluid it’s phase transition, changes in intracellular pH, and ribosomes rupture (Fig. 6) [24]. All these combined effects ultimately cause the complete or partial cellular components denaturation [119]. However, the HHP processing effectiveness depends on the pH, pressure, and temperature [6], [121].

In addition to enzyme inhibition, HHP processing destroys the microorganisms up to a safe level [35], [52], [118], [122], inactivates food pathogens and the oxidative shelf-life is more than twice as compared to the other NTTs [122]. The HHP processing is more applicable in food products that have high moisture content with flexible vacuum packaging. Orange juice, salsa, colourful products, flavoured fruit smoothies, ready-to-eat meats, purees are examples of HPP processed products [123].

It is evident that HHP processing has limited effects to achieve the desired enzyme inhibition in FJs [121], and its effect depends on the enzyme nature, the juices physicochemical properties, the medium pH, elevated temperature, ionic strength, total soluble solids, and so on [121]. It is possible to obtain high-quality products by applying the HHP processing at 100 MPa to 1000 MPa for a short duration typically ranging from a few seconds to several minutes, extending the shelf-life of various FJs (Table 5). These products are additive-free, with very slight alterations of their physical characteristics, sensory features, as well as nutritional properties [121]. On the other hand, the application of moderately HHP processing conditions, i.e., 200 to 600 MPa at ambient temperature, often results in the activation of the native enzymes (POD, PPO, and PME) in non-clarified FJs and fruit purees [24], [124], [125]. This can result in the development of undesirable modifications in FJs colour, flavour, texture, and nutritional quality during the processing and storage period [120]. However, these parameters can be controlled with proper refrigerated storage, appropriate packaging, the use of enzyme inhibitors, and oxygen scavengers. Therefore, with HHP optimization of FJs, it is vital to consider its effect on overall quality parameters, particularly on health-promoting bioactive components.

**Table 5:** HHP processing effect on the enzymes inactivation in fruit juices and purees.

**Fruit juice**	**Enzyme**	**Range given as (MPa/min/°C/ others, if any)**	**Max. inactivation at (MPa/min/°C/others, if any)**	**Results**	**References**
Apple	PPO	250–450.00/0–60.00/25.00–50.00	450/60/50 (91%)	• P-T interaction at > 400/>40 °C MPa	[34]
Kiwifruit juice	POD	200–600/0–30/10–50	600/30/50 (30%)	• Activation at 200 MPa/10 min/30 °C	[151]
Nectarine puree	PPO	400, 600/5/20–25/ (without or with ascorbic acid)	600/5/25/without ascorbic acid (60%)	• Activity retention of at 400 MPa/ in the presence of ascorbic acid	[118]
Peach juice	PPO	400–600/5–25/25	600/25/25 (79%)	• Activation (maximum107) at 400 MPa/15 min	[35]
Plum puree	PPO	400–600/0–5/20–25	20% (600/2.5/25)	• Non-significant effect for all treatments (P > 0.05)	[124]
Strawberry	PPO	400–800/5–15/18–22	800/15 (100%)	• Total deactivation at 800 MPa/15 min	[122]
Strawberry pulp	PPO	400–600/5–25/25	600/25/25 (48%)		[123]
	POD	400–600/5–25/25	600/5/25 (64%)	• Activation at 600 MPa > 5 min	[123]
Strawberry puree	PPO	100–690/5–15/24–90	690/5, 15/90 (23%)	• Deactivation from 16% to 23%	[152]
Watermelon juice	PPO	200–600/5–60/25	600/60/25 (88%)		[22]
	POD	200–600/5–60/25	600/60/25 (42%)		[22]
PPO: polyphenoloxidase; POD: peroxidase; PME: pectinmethylesterase; HHP: high hydrostatic pressure					

## 8 Conclusions and future perspectives

The NNTs employment potentially ensures to maintain the FJs safety, quality, and nutritional aspects for a long storage period. For this reason, there is a growing interest towards the use of NNTs in the current global markets, taking into account the actualregulatory authorities and manufacturers demand for FJs processing with a certain minimum level of quality and safety standards. However, a combination of these technologies with other methods may be required in order to achieve a synergistic enzyme inactivation effect. The primary action mode of NTTs consists in inducing structural changes in proteins tertiary and secondary structures to deactivate enzymes in FJs. Nevertheless, these modifications greatly depend on the food matrix processing intensity, and on the properties of enzymes and proteins present in the different FJs. Therefore, an appropriate optimization of both the equipment and the operation parameters involved in NTTs should also be done in order to guarantee an adequate control on the enzyme activities and a maximum efficiency. In addition, it is pivotal to consider the NTTs impacts on the safety and food quality. Despite the explored effects, NTTs have not been marketed for FJs processing industries. The major reasons could be the product acceptability, unavailability of advanced machinery, optimized process parameters and investment in scale-up process. Therefore, further efforts are required to commercialize these NTTs with an extensive array of desired benefits, taking into account the associated risks.

## Declaration of Competing Interest

The authors declare the following financial interests/personal relationships which may be considered as potential competing interests: Zhao Liqing reports financial support was provided by This work was supported by the National Key R&D Program of China (2021YFA0910800) and the Special Fund for Development of Strategic Emerging Industries in Shenzhen (JCYJ20190808145613154, KQJSCX20180328100801771).
